# Sample Preparation Strategies for the Effective Quantitation of Hydrophilic Metabolites in Serum by Multi-Targeted HILIC-MS/MS

**DOI:** 10.3390/metabo7020013

**Published:** 2017-03-30

**Authors:** Elisavet Tsakelidou, Christina Virgiliou, Lemonia Valianou, Helen G. Gika, Nikolaos Raikos, Georgios Theodoridis

**Affiliations:** 1 Laboratory of Analytical Chemistry, Department of Chemistry, Aristotle University of Thessaloniki, 54124 Thessaloniki, Greece; tsakeliz@hotmail.com (E.T.); cr_virgi@hotmail.com (C.V.); gtheodor@chem.auth.gr (G.T.); 2 Laboratory of Forensic Medicine & Toxicology, Department of Medicine, Aristotle University of Thessaloniki, 54124 Thessaloniki, Greece; gkikae@auth.gr (H.G.G.); raikos@auth.gr (N.R.)

**Keywords:** sample preparation, serum, endogenous metabolites, targeted metabolomics

## Abstract

The effect of endogenous interferences of serum in multi-targeted metabolite profiling HILIC-MS/MS analysis was investigated by studying different sample preparation procedures. A modified QuEChERS dispersive SPE protocol, a HybridSPE protocol, and a combination of liquid extraction with protein precipitation were compared to a simple protein precipitation. Evaluation of extraction efficiency and sample clean-up was performed for all methods. SPE sorbent materials tested were found to retain hydrophilic analytes together with endogenous interferences, thus additional elution steps were needed. Liquid extraction was not shown to minimise matrix effects. In general, it was observed that a balance should be reached in terms of recovery, efficient clean-up, and sample treatment time when a wide range of metabolites are analysed. A quick step for removing phospholipids prior to the determination of hydrophilic endogenous metabolites is required, however, based on the results from the applied methods, further studies are needed to achieve high recoveries for all metabolites.

## 1. Introduction

Liquid chromatography mass spectrometry (LC-MS) is one of the most popular techniques in metabolic profiling [1]. LC-MS metabolic profiling studies follow two paths as targeted or untargeted/holistic approaches. Untargeted methods generate large datasets that require extensive statistical analysis; they suffer from slow identification of metabolites and incomplete standardization [2]. This has resulted in increased interest for the development of tailor-made multi-analyte metabolomics methods, which are able to (semi)-quantify tens of analytes of specific interest in a single injection. Targeted LC-MS protocols provide solid, quantitative and unambiguous data [3,4,5,6].

However, quantitation of endogenous metabolites in biological fluids and particularly in serum has to overcome several issues. This is mainly due to pragmatic reasons, such as the lack of analyte-free matrix or Certified Reference Materials (CRMs). As a result, method validation including matrix effect and recovery is rarely described comprehensively. Matrix effect induces challenges in the analysis of endogenous analytes, as sample composition can differ among individuals, leading to errors. Apart from this, endogenous material builds up in the system (on the analytical column and ion source), decreasing the system’s lifetime and reducing sensitivity: the system is contaminated and the need for MS maintenance increases dramatically. Therefore, sample preparation plays a crucial part in accurate and precise quantitation by removing interferences and improving analytical performance and stability.

Ideally, a metabolic profiling method should be rapid, with minimum sample preparation, and should provide unbiased and robust results. Although sample preparation for LC-MS analysis is often minimal, it remains an error-prone analytical step, especially when dealing with complex biological matrices such as blood [7]. Typically, the minimum sample pre-treatment applied for blood prior to LC-MS analysis is a step of deproteinization [8,9,10,11,12,13,14], by the addition of an organic solvent. However, extraction recovery is based on the nature of the solvent used and the ratio of solvent/sample [15,16]. Potential problems can occur, such as losses due to poor solubility of compounds in the extraction solvent, solvent saturation effects, analyte co-precipitation with proteins, and poor chromatographic separation due to co-elution with endogenous compounds such as phospholipids [17]. In such simple preparation schemes a high amount of endogenous components is actually injected into the system together with the analytes of interest, resulting in poor accuracy if the analyte signal is affected. Endogenous phospholipids contained in serum (with approximately 17,000 different types of lipids and fatty acids being present*)* in significant levels are considered to be a real problem in HILIC (Hydrophilic Interaction Liquid Chromatography) systems. In HILIC mode, these species (Glycerophosphocholines (PC), sphingomyelins (SM), lysophosphatidylcholines (LPC), phosphatidylglycerols (PG), phosphatidylinositols (PI), and phosphatidylethanolamines (PE)) are eluted based on their ionizable moieties and appear in a wide range of retention times efficiency [18,19]. Even though phospholipids may not co-elute with the analytes, they can accumulate on the column and may elute in an unpredictable manner in subsequent analyses coverage [20]. This can cause ion suppression and poor reproducibility, compromising analytical accuracy. For this reason, phospholipids removal could be a step towards the elimination of matrix effect and errors in serum analysis. Unfortunately, the removal of phospholipids cannot be easily addressed due to the risk of removing the target analytes along with unwanted lipids.

Solid Phase Extraction (SPE) and Liquid–Liquid Extraction (LLE) are considered the most efficient methods for sample preparation [21,22]. In the analysis of hydrophilic endogenous metabolites, a combination of reversed phase SPE with HILIC chromatographic separation may prove a good selection [23]. Conventional SPE may involve lengthier sample preparation time and complicate method development. In applications which involve untargeted metabolite profiling, SPE can also introduce components leaching from the sorbent [24]. Hence the application of SPE should be considered with caution for such studies. Various commercially available sorbents offer additional selectivity and are able to extract molecules with specific characteristics [25,26]. Lately, novel SPE sorbents have been introduced that provide the removal of proteins and phospholipids from the sample in a single step. These contain either Zirconia or Titania, which strongly retain phosphate groups of phospholipids [18,27,28]. LLE, on the other hand, involves apolar organic solvents immiscible to water for the removal of lipid fractions including phospholipids [29,30].

The aim of the present study was to assess three different sample pretreatment methods for the removal of the endogenous lipophilic components of serum prior to Liquid Chromatography Tandem Mass Spectrometry (LC-MS/MS) analysis of hydrophilic metabolites. The typically performed protein precipitation (PPT) procedure was compared against three different sample pretreatment methods in achieving effective clean-up without sacrificing recovery. The advantages and disadvantages of conventional LLE methods and the combination of protein precipitation with SPE sample treatment prior to the application of a multi-analyte HILIC-UPLC-MS/MS method were investigated. The applied procedures were evaluated with regard to extraction efficiency, minimization of matrix effects and recovery. To the best of our knowledge this is the first investigation that studies these specific extraction methods with such a large number of metabolites with a targeted method which can provide quantitative results.

## 2. Results & Discussion

**Figure 1:**
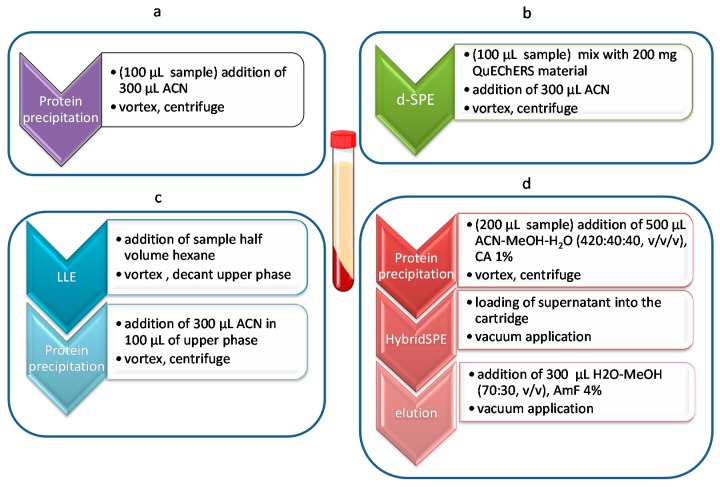
Schematic representation of the sample treatment protocols applied in the study indicating the steps needed prior to LC-MS/MS analysis, (**a**) standard protein precipitation procedure; (**b**) Dispersive-Solid Phase Extraction (d-SPE) (QuEChERS); (**c**) Liquid–Liquid Extraction (LLE); (**d**) HybridSPE.

The selected HILIC-MS/MS method is able to identify and quantify ca. 100 metabolites in a single injection. Of these metabolites, more than 50 were actually measured in serum samples. These metabolites include polar and ionisable molecules (negatively charged, positively charged or zwitterionic molecules) such as organic acids, amines, amino acids, purines, pyrimidines, carbohydrates, nucleosides and others (see Table 1). As these metabolites exhibit a wide variety of physicochemical properties, a generic extraction procedure is typically applied for LC-MS based metabolomics. Here the simple protein precipitation method often applied in our studies [31], and three alternate methods were compared. Figure 1 depicts schematically the standard PPT and the tree tested procedures. More details can be found in the Materials section.

### 2.1. Dispersive-SPE (QuEChERS)

QuEChERS (Quick Easy Cheap Effective Rugged Safe) procedure is a simple approach used for the extraction of various analyte classes; although QuEChERS was initially developed for pesticides analysis in food, it has lately found application in bioanalysis as well. To check the efficiency of the method, extraction recovery (R%) of the analytes was examined in a pooled serum sample by calculating the ratio of peak areas of the analytes obtained with and without QuEChERS step. With the application of QuEChERS, more than 50% of the target metabolites (24 out of 53) had lower recoveries below 70% in reference to the PPT method. However, eight metabolites showed improved signal response after QuEChERS clean-up, namely: thiamine, creatinine, choline, and certain compounds obtained through nutrition, such as theobromine, caffeine and cotinine. Overall this procedure was not considered beneficial for this application. Since our primary criterion was to detect at least 53 metabolites (detected by PPT), this method was not studied any further. However, other QuECheRS sorbents such as C18 or other newly introduced materials may be more effective in efficiently removing phospholipids without the loss of analytes, and this remains to be explored in further studies.

### 2.2. LLE

Another way to minimize matrix interferences is through the removal of the apolar fraction of serum by partitioning the extract in apolar solvents. LLE can provide clean extracts, however, it is time consuming and often involves the use of toxic organic solvents.

Results showed that the same number of compounds (*n* = 53) was detected in serum samples with and without LLE (simple PPT procedure). As above, in order to evaluate the benefit of this extra step, the analytes’ signals obtained with or without the extra step of LLE were compared. It was observed that eight compounds out of 53 had increased recoveries (R% above 130%): 3-methylhistidine, nicotinamide, hypotaurine, arginine, glutamine, uracil, benzoic acid and ornithine. Three metabolites showed significant decrease (R% below 70%) and the remaining 42 metabolites showed moderate decrease in response compared to the standard PPT method (R%: 70%–90%).

To evaluate any improvement due to the elimination of matrix effects, the slopes of standard addition calibration curves were compared versus the calibration curves obtained after the analysis of standards in neat solvent. For 40 out of the 53 detected analytes, calibration curves obtained after LLE showed slopes that differed to an extent higher than 10% from the corresponding curves in neat solvents. The corresponding number for calibration curves generated by simple PPT was lower (32 analytes showed a difference higher than 10% in comparison to curves from neat solvents), indicating a lower overall matrix effect. Furthermore, the use of LLE did not show improvement in the analysis of metabolites that showed significant matrix effect when using simple PPT (as arginine, glucose, lactate, isoleucine and others), so the additional LLE step was not selected for further application.

### 2.3. HybridSPE

Recently, several materials were made available in the market for removing proteins and phospholipids from blood samples in a single step. According to vendors, this process does not affect the recovery of target analytes. Here we applied HybridSPE, which, according to the manufacturer, provides the selective removal of phospholipids (via a Lewis acid-base interaction with Zirconia sites), while remaining non-selective towards a range of basic, neutral and acidic compounds.

**Figure 2:**
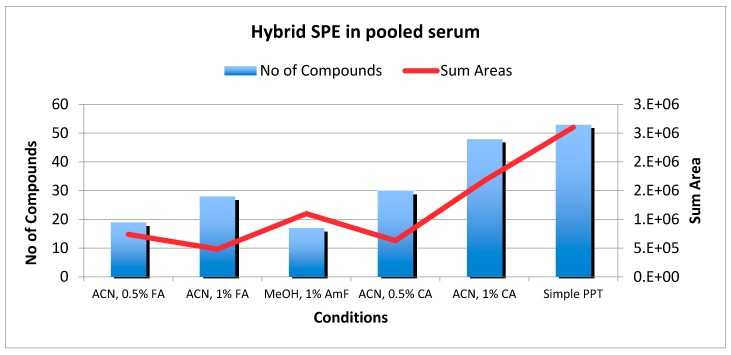
Plot showing the number of detected compounds (bars) and the sum of their areas (line) in the extract of pooled serum sample at different Hybrid SPE conditions.

The recovery of the analytes in a pooled serum sample was evaluated under different extraction conditions recommended by the HybridSPE manufacturer. As the set of analytes comprises both acidic, basic and zwitterionic compounds, a combination of modifiers was expected to be required. Initial trials used the minimum sample volume (100 μL) and protein precipitation on the SPE cartridge with acetonitrile (ACN) acidified with (a) 0.5% and 1% formic acid (FA); (b) 0.5% and 1% citric acid (CA) or (c) by buffering with 1% ammonium formate (AmF) in methanol (MeOH). It was found that recoveries of the target metabolites on HybridSPE cartridges were in general low in comparison to conventional PPT. More specifically, with the use of 0.1% FA, 33 of the compounds were retained on the cartridge and were not detected at all in the eluate. An increase in the FA concentration (0.5%) resulted in a slight increase in the number of the metabolites detected (24 retained), however, this still was a lower recovery in comparison to PPT. The use of CA improved recoveries for most of the metabolites, with 1% CA providing almost the same number of detected peaks (46) with PPT (53), but again with lower recoveries. As a quick measure, the number of analytes detected in the pooled serum and the sum of their intensities obtained under different conditions were compared. Figure 2 shows these numbers for all analytes detected for the different precipitation agents/conditions tested.

In studying particular metabolites groups, it was observed that a moderately acidic environment with FA was effective only for some nucleosides and nucleobases including thymine, thymidine, hypoxanthine, uracil and water soluble vitamins (nicotinamide, thiamine). It should be noted that organic acids (i.e., pyruvic acid) and amino acids (glycine, alanine, threonine, glutamine, etc.) were retained on the cartridge when FA was used in the precipitation agent, whereas this did not happen with 1% CA. This can be explained by the fact that amino acids act as chelating compounds to metals; CA is a stronger Lewis base that can displace and recover them from the cartridges. When 1% AmF in MeOH was used, again many of the analytes were not detected, whereas few analytes with basic properties such as proline, betaine and creatine could be detected (these metabolites were retained on the cartridge under acidic conditions). 3-Methyl-histidine, arginine and lysine seem to be strongly retained, thus they were not obtained with any of the abovementioned combinations.

**Figure 3:**
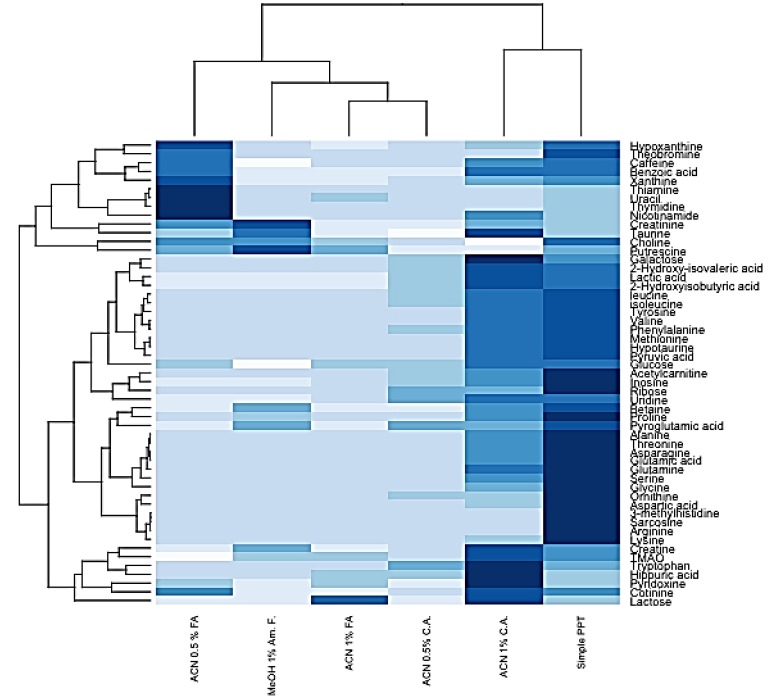
Pearson correlation heat-map indicating the response of the detected metabolites under PPT treatment and different HybridSPE conditions. Colour coding: signal response (peak area) increases from light to dark blue. Hierarchical clustering shows metabolites with similar trends with the applied protocols.

The signal of some metabolites was enhanced or decreased under different HybridSPE conditions. The Pearson correlation heatmap in Figure 3 indicates that there are groups of certain analytes that show similar trends under specific conditions, however, the responses for the majority of analytes are lower compared to the standard PPT.

Further efforts were focused on the enhancement of the recoveries of all metabolites without eluting phospholipids from the cartridge. Thus, a multi-step HybridSPE procedure was performed. As a first step, a higher sample volume was used, namely 200 μL. Then, extraction was performed using acetonitrile, 1% CA. This protocol was improved further by the addition of a second elution step at a higher pH, with the aim of eluting retained compounds such as arginine, lysine, 3-methylhistidine, thiamine, choline, etc. Different ratios of MeOH, H_2_O and ACN with various concentrations of ammonium formate were examined. Optimum results were obtained with 70:30 *v*/*v* H_2_O: ACN, 4% AmF. To find the optimum solvent volume for each step, additional elutions were performed and the eluents were analysed to determine the retained fraction of each metabolite. At this point it should be noted that, with the composition of the finally selected solvent, protein precipitation was not effective. Thus protein precipitation was performed as a separate step before loading onto the cartridge. Finally, the optimum recoveries were obtained with the following protocol: 200 μL of sample was mixed with 500 μL of a mixture of 84:8:8 (*v*/*v*/*v*), ACN/H_2_O/MeOH, containing 1% CA. The resulting mixture was vortex-mixed for 1 min and centrifuged at 4 °C for 5 min. The supernatant was transferred onto the Hybrid SPE cartridge and vacuum was applied. A second elution step was performed with 300 μL 70:30 H_2_O: ACN, 4% AmF. The combined eluent was directly analysed by LC-MS.

**Figure 4:**
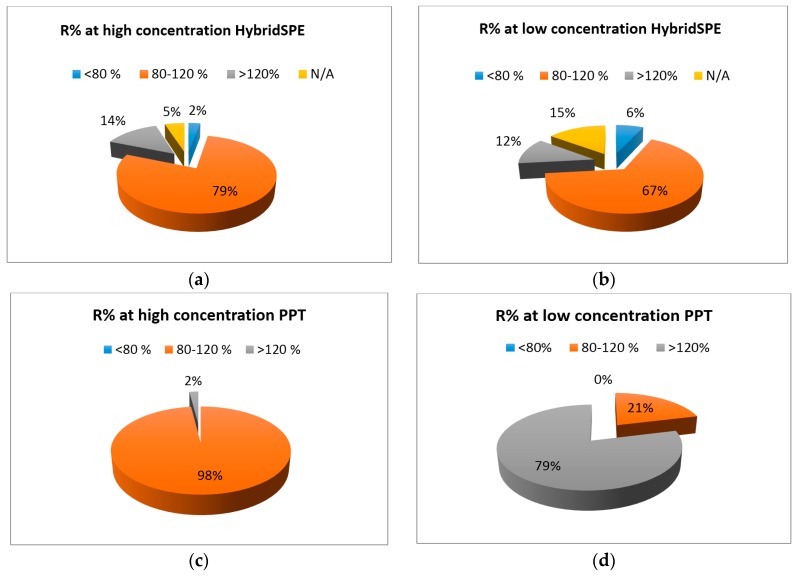
Distribution of recovery values for the 53 analytes under optimum HybridSPE conditions at (**a**) high and (**b**) low concentration. The respective recovery values for simple PPT are shown in (**c**) and (**d**) for high and low concentration in spiked pooled serum.

Recovery of analytes under the optimum conditions of HybridSPE was evaluated at both low and high concentration levels. The distribution of the recovery values (R%) obtained for all 53 target metabolites at low and high concentration levels are illustrated in Figure 4a,b.

Of the target metabolites, 79% and 69% exhibited satisfactory recoveries between 80 and 120% for the high and low concentration levels, respectively. Lactic acid, glucose, proline and 3-methyl histidine exhibited recovery lower than 80% in the low concentration, while only theobromine was found with recovery lower than the acceptable limit at the high concentration pooled sample. Arginine, inositol, isoleucine methionine and ornithine showed recoveries above the upper limits (>120%) in both low and high concentration levels. In comparison to recovery values obtained by the standard PPT method (which were found to range from 88% to 121%), it can be seen that there is a compromise when HybridSPE extraction procedure is applied (Figure 4). All analytes with an exception of one, had recoveries within the acceptable range of 80%–120% in the simple PPT procedure.

The efficiency of the applied protocol was also examined in terms of the amounts of phospholipids found in the extracts and the overall effect on analytes responses (in comparison to standard solutions, matrix effect—ME ).

**Figure 5:**
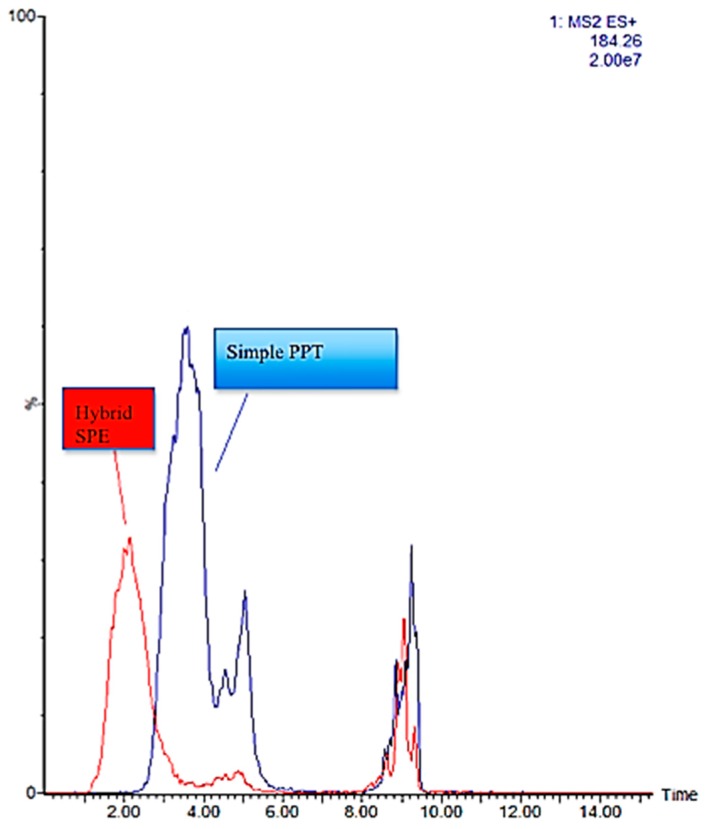
Overlay extracted ion chromatograms (*m*/*z * 184) from full scan analysis of samples after PPT and HybridSPE procedures.

In Figure 5, the extracted ion chromatogram (EIC) of *m*/*z* 184.6 from a full scan analysis of the pooled sample treated both with the conventional PPT and HybridSPE method is provided. Glycerophosphatidylcholines and sphingomyelins, particularly ceramidephosphocholines, experience the same fragmentation pattern in positive ionization with the product ion *m*/*z* 184.0730 as the most characteristic fragment for both families, which corresponds to the phosphorylcholine moiety. This trace in MS or MS/MS acquisition function is used to monitor the total phosphatidylcholine content of a sample. As can be seen in the Figure, this trace is significantly decreased in the extract of HybridSPE in a time window from 1 to 6 and from 8 to 10 min, indicating the removal of phosphatidylcholines.

Matrix Effect (ME) was calculated asmatrix factor (MF), for every analyte detected in the pooled serum under the optimum HybridSPE protocol and was compared with those from the standard PPT procedure. In Table 2, the matrix factor is given for all detected analytes with the two sample preparation methods.

It was found that eight analytes showed an increased signal of 20% (MF > 1.2) and seven showed a decreased 20% (MF< 0.8) in HybridSPE, whereas these numbers were 11 and six for PPT. Based on this it was concluded that the SPE process did not significantly suppress matrix effects that occur when simple PPT is performed. Improvement was observed for specific analytes, e.g., matrix factor was reduced for creatine, galactose, tryptophan, proline, glucose and inosine.

Analytical precision was found satisfactory for the majority of the analytes. In total, 42 out of the 53 analytes had RSD% below 10% and the mean RSD% of all analytes was 9%.

In Figure 2, the applied protocols are illustrated. In general, the PPT procedure is the most time and cost effective method compare to LLE, dispercive-SPE (d-SPE) and HybridSPE. Both PPT and d-SPE are quick methods (one step) compared to LLE (two steps) and HybridSPE (three steps). Additionally, consumables are needed for the dispersive-SPE and HybridSPE procedures, increasing the cost of the analysis.

## 3. Conclusions

In metabolomics, the diversity of the structure and physicochemical properties of the analysed molecules impel us to apply sample preparation methods as generic as possible. Moreover, while for single analyte assays proper sample clean-up and optimization of extraction parameters might be straight forward, for multi-targeted assays this is a real challenge. On top of that, the high number of samples analysed in metabolomic studies can negatively affect the analytical system and its performance due to matrix accumulation during runs. As a result, a minimal sample preparation procedure, although highly attractive, may compromise the accuracy and robustness of the obtained results, introducing non-anticipated biases. In such applications a quick and effective clean-up must be combined with good analyte recovery and metabolome coverage. The literature includes papers that describe studies of extraction utilizing untargeted LC-MS mode. In this mode, peak heights of unknown features are compared. The present paper is the first investigation that studies a large number of endogenous metabolites using targeted, quantitative analysis and sample preparation by these specific modes.

Here we show that an additional sample preparation step, using novel materials under proper conditions, can improve analytical performance for certain analytes. It was found that in order to obtain satisfactory recovery, more than one pre-treatment step is required. Thus, a balance between time and effort, cost and the obtained benefits should be considered. It was found that an LLE step applied prior to PPT can remove phospholipids; however, there is some loss of certain analytes. HybridSPE can be a good solution when metabolites of a similar nature are to be determined, e.g., a panel of short chain fatty acids. In the present study, where various hydrophilic metabolites were of interest, multiple treatment steps were needed. A quick d-SPE using a sorbent that does not retain polar analytes would provide an ideal solution for this type of analysis.

## 4. Experimental

### 4.1. Reagents and Materials

Acetonitrile (Carlo Erba, Val de Remil, France) and n-hexane (CHROMASOLV, Schnelldorf, Germany) of LC-MS grade were used. Ultra-pure Water (18.2 MΩ cm) was obtained by Millipore water purification system (Merck, Darmstadt, Germany). Ammonium formate of LC-MS grade, ammonium hydroxide, formic acid and citric acid of analytical or higher grade (Sigma Aldrich, Gillingham, Dorset, UK) were used as additives. All metabolite standards of analytical grade were obtained from various vendors. 2 mL Bond Elut QuEChERS Dispersive Kit PN 5982-5022 (Agilent, Technologies Santa Clara, CA, USA) and HybridSPE-Phospholipid Ultra Cartridges (30 mg, volume 1 mL, Sigma-Aldrich/Supelco, Bellefonte, PA, USA) were used. An Acquity BEH Amide Column (2.1 mm × 150 mm, 1.7 μm) analytical column together with an Acquity UPLC Van-Guard precolumn was used for chromatographic separations.

### 4.2. Analytical System Parameters

The HILIC-UPLC-MS/MS method used was developed by our group and details on its development and validation can be found in the literature [32]. An Acquity UPLC System with a XeVo TQD LC-MS system (Waters Corporation, Milford, CT, USA) was used. HILIC mode was applied with a gradient elution of 30 min starting from a 100% solvent A (ACN/water 95:5, *v*/*v*, 10 mM AF) 4 min isocratic step, to 40% solvent B (water/ACN 70:30, *v*/*v*, 10 mM AF) in 21 min and finally to 85% B in 5 min. Temperature was set at 40 °C and the flow rate was at 0.5 mL/min while injection volume was set at 5 μL.

ESI source operated in polarity switching mode with capillary voltage at +3.5 kV or −3.5 kV and block and desolvation temperature at 150 °C and 350 °C, respectively. Desolvation gas flow rate was at 650 L/h and cone gas was at 50 L/h.

Detection was performed in Multiple Reaction Monitoring (MRM) mode for 101 traces in specific time windows with dwell times optimised for specific compounds in order to increase sensitivity. For full scan acquisition, cone voltage was set to 20 V, mass scan range was 50 to 900 amu and scan time was set at 0.4, for both positive and negative ionization modes.

### 4.3. Sample Preparation Methods

For the removal of interfering lipid fraction of serum samples, three different pretreatment clean-up methods were applied and compared against a conventional protein precipitation step with 1:3 (*v*/*v*), serum/ACN (Figure 2). This protocol has been applied in our previous studies [33]. Based on Matuszewski et al. [34], matrix effects should be investigated in biofluid samples from at least five different sources. In this study on recoveries and relative matrix effect assays a pooled serum sample was prepared from five healthy adult human subjects. A quality control sample (QC) was prepared as a representative sample by mixing equal volumes of each serum sample.

For the simple PPT method, 100 μL of serum was vortex-mixed with 300 μL ΑCN for one minute, centrifuged at 7000 g and 4 °C for 10 min and 5 μL of the clear supernatant was directly injected into the system (Figure 2).

Quantitation was performed by standard addition: QC aliquots (50 μL) were spiked with standard mixtures at five concentration levels (150 μL of standard mix in 130:10:10 ACN: MeOH: H_2_O) see Supplementary Information Tables S1 and S2). Based on the standard addition calibration curve, the concentration of the endogenous metabolites was calculated as intercept/slope.

Recovery of conventional PPT procedure and HybridSPE was evaluated in two different concentration levels (low and high, see Supplementary Information Tables S1 and S2). QC serum samples were spiked before and after extraction.

Chromatographic peak areas of standard mixtures were compared before and after treatment. Matrix effect for endogenous compounds was assessed by spiking eluted serum samples with the standard mixtures and using the equation below in order to calculate the matrix factor.

Recovery = (Area (prior to extraction spiking QC) − Area (post extraction spiking QC)) × 100%.

Matrix Factor = ((Area (post extraction spiking QC) − Area (QC))/Area (standard).

For all experiments analysis of the serum extract was performed in two replicates.

Full scan chromatograms were acquired for extracts under the different extraction protocols to examine for lipid traces in the background.

#### 4.3.1. Dispersive SPE

Α dispersive SPE (d-SPE) protocol was applied with a QuEChERS dispersive Kit. The approach includes two steps: (1) a buffering and initial extraction step with acetonitrile where protein precipitation also takes place in the case of serum and (2) a second clean-up step with MgSO_4_ and a sorbent to remove water and undesired co-extracted lipids, respectively. Here the introduction of a d-SPE-QuEChERS clean-up step in the standard PPT procedure was investigated using MgSO_4_ and PSA (primary secondary amine). From the 2 mL tubes containing 50 mg PSA and 150 mg MgSO_4_, 10 mg were transferred to a 1.5 mL vial where 100 μL of serum were added and mixed with 300 μL ΑCN, vortexed for 1 min and finally centrifuged at 7000 g and 4°C for 10 min (Figure 2). The same procedure was followed for the standard addition application (five points calibration curve).

#### 4.3.2. LLE Delipidation

Delipidation of serum samples before other treatment was also tried by Liquid–Liquid Extraction (LLE) with a non-polar solvent. 1 mL of serum sample was vortex-mixed with 500 μL of n-hexane, for 1 min. Centrifugation followed at 6000 *g* for 5 min and the upper layer was discarded. Then 100 μL of the lower aqueous layer were vortex-mixed with 300 μL of ΑCN for one minute and finally centrifuged again at 7000 *g*, 4 °C for 10 min (Figure 2). Five μL of the clear supernatant were injected into the system. The same procedure was followed for spiking serum samples in order to construct a five point calibration curve for standard addition approach.

#### 4.3.3. HybridSPE

Solid Phase Extraction with a novel Zirconia coated silica based material (HybridSPE^®^-Phospholipid Technology) for the removal of phospholipids and proteins was alternatively applied. HybridSPE-Phospholipid cartridges were used under various conditions. Based on manufacturer guidelines three different modifiers can be used to aid protein precipitation and hinder the retention of analytes for the Zr-Si sorbent together with the phospholipids: formic acid (FA), citric acid (CA) and ammonium formate (AmF). Formate and citrate ions are strong Lewis bases, which compete with the acidic analytes on the Zr sites and inhibit their retention, whereas acidic conditions introduced by FA deactivates residual silanols and prevents the retention of basic analytes. Citrate is a stronger Lewis base compared to formate, and can thus aid in the recovery of even the most highly interacting analytes with Zr sites (analytes with chelating functional groups) without disturbing phospholipid binding. Ammonium cation is used since it can act as a competing ion for disrupting weak cation exchange interactions with residual silanols of the silica surfaces, thus ammonium formate is recommended in combination with citrate and formate. Substitution of ACN with MeOH can aid in the inhibition of secondary HILIC interactions which may occur between basic/neutral analytes of interest and the silica surface, while water is necessary at a minimum ratio of 25%. Ammonium hydroxide is also applied to achieve alkaline conditions for the elution of all components and phospholipids [35].

Citric acid was used as an extraction agent with or without prior conditioning (400 μL 0.5 or 1% CA in ΑCN). Parameters such as sample volume, additives and solvents were tested to obtain effective clean-up and high recoveries for all of the endogenous hydrophilic analytes. First trials were carried out with 100 μL sample volume and protein precipitation with 300 μL of MeOH or ΑCN, enriched with additives, such as 0.5% or 1% FA, 1% AmF and 0.5%–3% CA. Loading of the aliquot onto the cartridge and vacuum application followed, according to manufacturer's recommendations. Thereafter, extraction protocol was optimized in order to avoid limitation related to losses of endogenous compounds. Finally, the optimum protocol (Figure 2) was evaluated and the matrix effect, recovery and repeatability of the method were assessed.

## Figures and Tables

**Table 1:** Target metabolites per class of compounds determined in serum by the applied analytical method.

Class	Metabolites	
**Purine/Pyrimidine/Pyridoxine Derivatives**	Hypoxanthine	Theobromine
	Caffeine	Thymidine
	Cotinine	Uracil
	Inosine	Uridine
	Nicotinamide	Xanthine
	Pyridoxine	
**Acids**	Benzoic acid	Hippuric acid
	2-Hydroxyisovaleric acid	Lactic acid
	2-Hydroxyisobutyric acid	Pyroglutamic acid
	g-Aminobutyric acid	Pyruvic acid
**Polyamines**	Putrescine	
**Amino acids medium polarity**	Alanine	Proline
	Glycine	Tryptophan
	Leucine	Tyrosine
	Methionine	Valine
	Phenylalanine	Ιsoleucine
**Charged Amino acids /Amines**	Acetylcarnitine	Lysine
	Arginine	Thiamine
	Betaine	Trimethylamine-n-oxide
	Choline	
**Polar Chain, Ionisable Amino acids /Derivatives**	Aspartic acid	Glutamic acid
	Asparagine	Ornithine
	Creatine	Threonine
	Creatinine	Sarcosine
	Glutamine	Serine
	Hypotaurine	Taurine
	3-Methylhistidine	
**Carbohydrates**	Galactose	Lactose
	Glucose	Ribose

**Table 2:** Comparison of the final selected HybridSPE protocol with the standard PPT in terms of matrix effect (ME). Retention time of target metabolites in bold indicates elution of analyte in the area of phospholipid elution region.

Analyte	Rt (min)	MF^*^	
		Conventional PPT	HybridSPE
2-Hydroxyisobutyric acid	7.53	1.20	0.97
2-Hydroxyisovaleric acid	5.4	1.00	1.02
3-methylhistidine	19.32	1.13	1.22
Acetylcarnitine	**14.22**	0.96	0.92
Alanine	15.91	1.12	1.09
Galactose	12.08	0.83	0.86
Arginine	21.84	2.62	1.75
Asparagine	18.29	1.04	1.04
Aspartic acid	21.37	0.99	0.80
Betaine	12.11	0.73	0.79
Caffeine	0.87	0.77	0.80
Choline	6.72	0.95	0.91
Cotinine	**1.10**	0.96	0.87
Creatine	**16.10**	0.73	0.85
Creatinine	**4.65**	0.92	0.77
g-aminobutyric acid	16.69	1.01	0.88
Glucose	**14.24**	0.62	1.07
Glutamic acid	20.83	1.05	1.07
Glutamine	17.65	1.32	1.31
Glycine	16.86	1.36	1.29
Hippuric acid	**9.2**	1.10	1.14
Hypoxanthine	**4.71**	0.89	0.91
Inosine	**8.97**	1.24	1.07
Ιsoleucine	13.27	2.25	1.87
Lactic acid	11.12	0.48	0.68
Leucine	12.82	1.49	1.42
Lysine	22.3	1.47	1.27
Methionine	14.00	1.09	0.88
Valine	14.26	0.98	1.00
Ornithine	22.52	1.42	0.74
Phenylalanine	12.54	1.22	1.19
Proline	**14.24**	0.81	1.09
Putrescine	20.84	1.00	0.83
Pyridoxine	**2.00**	1.00	0.89
Pyroglutamic acid	14.78	1.07	1.02
Pyruvic acid	7.06	0.89	0.84
Ribose	**4.25**	0.87	0.93
Sarcosine	15.1	0.90	1.04
Serine	17.78	1.21	1.02
Taurine	14.24	1.04	0.86
Theobromine	**1.15**	0.93	0.44
Thiamine	11.57	1.12	1.06
Threonine	16.62	1.13	1.11
Thymidine	**1.91**	1.02	0.64
Trimethylamine-*n*-oxide	12.64	1.02	1.01
Tryptophan	12.66	1.18	1.10
Tyrosine	14.42	1.01	1.05
Uracil	1.82	1.13	0.83
Uridine	**4.56**	0.85	0.96
Xanthine	7.28	1.10	1.04
Benzoic acid	1.63	0.20	0.28
Hypotaurine	15.62	1.32	1.24
Lactose	18.31	1.01	1.16
Nicotinamide	1.16	1.10	0.86

* Matrix effect is expressed as matrix factor (MF)

## Supplementary Materials

The following are available online at www.mdpi.com/2218-1989/7/2/13/s1, Table S1: Metabolites determined in serum by the applied LC-MS/MS method divided in three groups based on their intrinsic concentration in blood, Table S2: Spiking concentrations for the standard addition calibration curves and for the assessment of recovery.
