# Supplementary material for: Sample Preparation Strategies for the Effective Quantitation of Hydrophilic Metabolites in Serum by Multi-Targeted HILIC-MS/MS
Source: Metabolites. 2017 Mar 30;7(2):13. doi: 10.3390/metabo7020013 (PMC5487984; doi:10.3390/metabo7020013)
Supplement: Supplementary file 1 [file metabolites-07-00013-s001.pdf]

Supplementary Materials: Sample Preparation Strategies for the Effective Quantitation of Hydrophilic Metabolites in Serum by Multi-Targeted HILIC-MS/MS

Elisavet Tsakelidou, Christina Virgiliou, Lemonia Valianou, Helen G. Gika, Nikolaos Raikos and Georgios Theodoridis

Table S1. Metabolites determined in serum by the applied LC-MS/MS method divided in three groups based on their intrinsic concentration in blood.

Group A	Group B	Group C
Hypoxanthine	Glycine	Lactic
Theobromine	Xanthine	Aspartic acid
Caffeine	Taurine	Lysine
Benzoic acid	Galactose	Uracil
Thiamine	2-Hydroxy-isovaleric acid	Glucose
Thymidine	2-Hydroxy-isobutyric acid	
Nicotinamide	Leucine	
Creatinine	Isoleucine	
Acetylcarnitine	Tyrosine	
Ribose	Valine	
Choline	Phenylalanine	
Betaine	Methionine	
Putrescine	Inosine	
Creatine	Hypotaurine	
TMAO	Pyruvic acid	
Pyridoxine	Proline	
Cotinine	Uridine	
Lactose	Pyroglutamic acid	
	Alanine	
	Asparagine	
	Threonine	
	Glutamine	
	Glutamic acid	
	Serine	
	Ornithine	
	Tryptophan	
	3-Methylhistidine	
	Sarcosine	
	Arginine	
	Hippuric acid	

Table S2. Spiking concentrations for the standard addition calibration curves and for the assessment of recovery.

		Concentrations in serum		
	**spiking**	**Group A (µg/mL)**	**Group B (µg/mL)**	**Group C (µg/mL)**
**5 point curves**	Level 1	0.08	0.72	3.80
	Level 2	0.20	1.80	9.50
	Level 3	0.50	4.50	23.80
	Level 4	1.00	9.00	47.50
	Level 5	1.20	10.80	57.00
**LQC**	low level spiking	0.10	0.90	4.75
**HQC**	high level spiking	1.00	9.00	47.50
